# Pigment Epithelium-Derived Factor as a Possible Treatment Agent for Choroidal Neovascularization

**DOI:** 10.1155/2020/8941057

**Published:** 2020-03-06

**Authors:** Lei Xi

**Affiliations:** Division of Experimental Vitreoretinal Surgery, Centre for Ophthalmology, University of Tübingen, Schleichstrasse 12/1, 72076 Tübingen, Germany

## Abstract

Choroidal neovascularization (CNV) is a sight-threatening disease and is characterized by the formation of pathological neovascularization in the choroid which extends into the subretinal space. Exudative age-related macular degeneration (AMD) is the formation of CNV in the macular area which leads to irreversible blindness. Continuous leakage and hemorrhage of the CNV lesion may eventually result in scarring or later fibrosis, which could result in photoreceptor cell atrophy. The current strategy for treating CNV is the use of antivascular endothelial growth factor (VEGF) agents. Many studies have demonstrated the efficacy of intravitreal anti-VEGF therapy. Other studies have also reported the side effects of single anti-VEGF treatment. And long-term inhibition of a single system may result in collateral damage to other visual elements. Pigment epithelium-derived factor (PEDF) is a 50 kDa protein that was first isolated from the conditioned medium of human RPE cells. PEDF has both antiangiogenesis and neuroprotective functions for photoreceptor cells. It may be a potential ocular antiangiogenic agent. This review outlines the distribution of PEDF in the eye, the mechanism of antiangiogenesis, the protective effect on the retina, and the relationship between PEDF and VEGF.

## 1. Introduction

Choroidal neovascularization (CNV), particularly when associated with age-related macular degeneration (AMD), is one of the leading causes of severe vision loss in the elderly. The reason for the formation of CNV is still not clear. It is recognized that the abnormal increase of the vascular endothelial growth factor (VEGF) plays a central role in the development of CNV [1]. In animal studies, increased expression of VEGF in the RPE can lead to CNV [2–4]. The most common causes of CNV are AMD and pathologic myopia, which have abnormalities or defects of Bruch's membrane (BM) [5, 6]. BM separates the vascular choroid from the avascular outer retina. Any damage to the BM in the CNV can result in retinal edema and subretinal hemorrhage. Studies have shown that the rupture of BM with laser photocoagulation in rats results in CNV [7, 8]. New blood vessels are easy to leak. Subretinal CNV leakage results in excessive extracellular matrix deposition, fibrosis, and scar formation. Ultimately, normal tissue is replaced with permanent scar tissue, which leads to decreased organizational function. Previous studies showed that PEDF has an antifibrotic effect [9–12]. Fibrosis occurs in the late stage of extracellular matrix, leading to permanent loss of vision.

The pigment epithelium-derived factor (PEDF) is a member of the serine protease inhibitor supergene family [13]. It was first purified from the conditioned medium of human retinal pigment epithelial (RPE) cells as a factor that induces neuronal differentiation of cultured Y79 retinoblastoma cells [14]. PEDF is a multifunctional protein, and it has antiangiogenic, antioxidative, neuroprotective, and neurotrophic functions. Animal experiments have confirmed the functions of PEDF in the eye. Recently, anti-VEGF agents have been used in the management of CNV. Although antiangiogenic therapy can inhibit new vessel growth, exudation or bleeding of CNV is difficult to absorb and affects the function of photoreceptor cells. Anti-VEGF treatment has many side effects, such as thrombotic microangiopathy [15], deposition on the retinal vessels [16], and increased mortality in AMD patients after acute myocardial infarction (MI) [17]. These side effects have been found in animal experiments. PEDF is a promising agent for the treatment of ocular neovascular diseases. However, there has been less research on the antifibrotic effect of PEDF in the eye. If PEDF can be used clinically in the future, treatment of choroidal neovascularization will improve vascular leakage and protect photoreceptor cells.

This review describes the expression and distribution of PEDF in the eye, research status, and possible mechanisms of its anti-CNV functions. The possibility of combining PEDF with common anti-VEGF drugs is also introduced.

## 2. Expression of PEDF in the Eye

Expression of PEDF protein in the eye has been investigated in both rats and humans. Different studies have different findings. Ogata et al. found that mRNA of PEDF was expressed in different cell types of normal rat eyes, such as corneal epithelial cells, corneal endothelial cells, lens epithelial cells, ciliary epithelial cells, the ganglion cell layer, and RPE cells [18]. Renno et al. investigated changes in expression of the PEDF in the retina over the course of CNV's development in the rat model of laser-induced CNV. They observed the downregulation of PEDF's expression (weak immunoreactivity) through the CNV regions and adjacent flanking areas, suggesting that the loss of PEDF plays a permissive role in the formation of CNV. Also, they detected the vitreous levels of PEDF protein during the formation of laser-induced CNV by Western blot analysis. There is an increase in PEDF vitreous protein levels during the first week after induction of CNV, but no difference was found comparing the normal nonlasered rats and sham-lasered rats. The special finding is that PEDF immunostaining in the normal rat retinal layers localized mainly to the outer nuclear layer (ONL), which is the most avascular layer of the retina [19]. Other studies also found that the PEDF secretion pattern from the RPE cells is predominantly apical, and the interphotoreceptor matrix around the RPE microvilli is a major reservoir of PEDF [20, 21].

Karakousis et al. used immunocytochemistry to localize the PEDF in fetal and adult eyes with a polyclonal antibody (pAb) against amino acids 327-343 of PEDF. They found that pAb anti-PEDF labeled retinal RPE granules, developing cones, some neuroblasts, and many cells in the ganglion cell layer (GCL) in developing retinas (7.4 to 21.5 fetal weeks (Fwks)). In adult retinas, immunolabeling with pAb anti-PEDF demonstrated the protein in the rod and cone cytoplasm and nuclei of rods but not cones. Cells in the INL and GCL, choroid, corneal epithelium and endothelium, and ciliary body were also pAb PEDF-positive [22]. Their result demonstrated that the PEDF-positive band in the outermost retina was in the cytoplasm of the differentiating photoreceptors and not the interphotoreceptor matrix (IPM). Photoreceptors and RPE may secrete PEDF into the IPM.

There are also reports on PEDF level changes in the eyes of human CNV and AMD patients. Bhutto et al. examined the localization and relative levels of VEGF and PEDF in aged human choroid and AMD by immunohistochemical staining. They found that the most prominent sites of VEGF and PEDF localization in an aged control choroid were RPE Bruch's membrane-choriocapillaris complex including the RPE basal lamina, intercapillary septa, and choroidal stroma [23]. The PEDF levels in the RPE, RPE basal lamina, Bruch's membrane, and choroidal stroma were significantly lower in AMD subjects compared to aged control subjects. Tong et al. examined the aqueous humor levels of VEGF and PEDF in active CNV secondary to AMD and pathologic myopia. They found that the aqueous humor levels of both VEGF and PEDF were significantly increased in patients with CNV of AMD and CNV of pathologic myopia, when compared with the senile cataract controls [24]. Holekamp et al. collected undiluted vitreous from eyes of patients with CNV and age-matched control eyes of patients and examined the vitreous PEDF and VEGF by Western blot analysis and enzyme-linked immunosorbent assay (ELISA). They found that PEDF was deficient in the vitreous of patients with CNV due to AMD [25]. So, they hypothesized that loss of PEDF might create a permissive environment for CNV patients with AMD. The expression of PEDF and VEGF within the tissues of subfoveal fibrovascular membranes was investigated. PEDF and VEGF were strongly expressed in endothelial cells and RPE cells located in perivascular areas in active choroidal neovascular membranes (CNVMs). However, in quiescent CNVMs, PEDF and VEGF were weak or not detectable in the new vessels [26]. So, the levels of PEDF and VEGF may depend on the clinical status of the subfoveal fibrovascular membranes.

## 3. Mechanism of PEDF Inhibiting Neovascularization

### 3.1. Inhibition of the VEGF Pathway

How PEDF controls the growth of new blood vessels is not well understood. Recent treatments for ocular neovascularization are mainly anti-VEGF. Members of this family include VEGF-A, VEGF-B, VEGF-C, VEGF-D, VEGF-E, and VEGF-F. VEGF-A is typically referred to as VEGF. The abnormal expression of VEGF-A has become a focal point of current research in retinal and choroidal neovascular diseases [27].

The interactions between PEDF, a potent angiogenic inhibitor, and VEGF, an angiogenic stimulator, are largely unclear. Zhang et al. demonstrated that PEDF significantly decreased VEGF expression in both retinal capillary endothelial cells (RCEC) and Müller cells in oxygen-induced retinopathy rats. Also, PEDF decreased VEGF promoter activity under hypoxic conditions but not under normoxic conditions [28]. There may be a balance between PEDF and VEGF levels. Gao et al. demonstrated an increased VEGF and decreased PEDF in the ischemia-induced retinal neovascularization rat model by using Northern blot analysis. They found that the time course of the PEDF downregulation is consistent with the increase of VEGF expression, suggesting that an unbalance between angiogenic stimulators and inhibitors may contribute to retinal neovascularization [29].

PEDF downregulating VEGF expression might partially be via inhibition of mitogen-activated protein kinase- (MAPK-) mediated hypoxia-inducible factor 1 (HIF-1) activation and competes with VEGF binding to RCEC and VEGFR-2. They suggested that the competitive blockade of the VEGF-RCEC and VEGF-VEGFR-2 binding may be responsible for the PEDF effects on VEGF-induced permeability and angiogenesis [28]. Cai et al. demonstrated that the inhibitory effect of PEDF on VEGF-induced angiogenesis results from enhancing the *γ*-secretase-dependent cleavage of the C-terminus of VEGFR-1, which in turn inhibits VEGFR-2-induced angiogenesis. In addition, PEDF was also able to regulate the phosphorylation of VEGFR-1, which itself can regulate VEGFR-2 signaling [30]. However, Johnston et al. suggested that the interaction between PEDF and VEGFR-1 or VEGFR-2 might promote the receptors' internalization and/or degradation to limit VEGF function and then block angiogenetic activity [31]. Kanemura et al. concluded that PEDF enhanced *γ*-secretase activities leading to cleavage of VEGF receptor-1 and VEGF receptor-2 [32].

Wang et al. investigated the *in vitro* effects of PEDF on primary cultured human choroidal endothelial cells (CECs). They found that PEDF had little effect on normal CEC proliferation and migration. PEDF suppressed the proliferation and migration of VEGF-induced choroidal capillary endothelial cells [33]. PEDF appeared to have blocked abnormal neovascularization without overt harm to established retinal vessels. This suggested that the response to PEDF treatment may vary with cell types [34].

### 3.2. Receptor-Mediated and Other Signaling Pathways

The formation of blood vessels is an intricate process. The process needs participation of endothelial junction proteins, tube formation factors, and vessel stabilization molecules. Targeting these molecules during the formation of neovascularization may have advantages in antiangiogenic strategies [35]. PEDF's action on endothelial cells is also likely to be receptor mediated but perhaps by way of a different receptor [34].

Chen et al. examined the molecular actions of PEDF on Human Umbilical Vein Endothelial Cells (HUVEC). They found that induction of apoptosis in human endothelial cells by PEDF is through the activation of both the receptor-mediated and mitochondria-mediated pathways with activation of p38 followed by cleavage of caspases 3, 8, and 9. The result suggests that the apoptotic effects of PEDF are through multiple pathways and caspase dependent [36]. The nonintegrin 37/67 kDa laminin receptor (LR) is a new PEDF receptor. The antiangiogenic activity of PEDF is at least partially mediated by binding LR. Modulating LR activity could provide an attractive option for treating angiogenesis-dependent disease [37].

Fas ligand (FasL) was induced by thrombospondin-1 (TSP1) and PEDF on endothelial cells, which destruct newly formed vessels marked by angiogenic stimuli that induced Fas. Volpert et al. suggested that the antiangiogenic activity of TSP1 and PEDF was dependent on the dual induction of the Fas and FasL pathways. They found that TSP1 and PEDF increased the surface display of Fas by using flow cytometry, and the dynamics of FasL expression were consistent with the kinetics of endothelial cell killing by TSP1 and PEDF [38]. Haribalaganesh et al. demonstrated that PEDF inhibits erythropoietin- (EPO-) induced proliferation, migration, tube formation, and permeability in goat retinal microvascular endothelial cells (GRECs). Further, PEDF inhibits the EPO-induced PI3K/Akt signaling in endothelial cells [39].

Macrophages have been demonstrated to play a proangiogenic role in retinal pathological vascular growth. The macrophages express high levels of VEGF and increase VEGFR expression in HUVEC. The macrophages might stimulate endothelial proliferation, thus contributing to the generation of neovascular tufts (NVTs). PEDF works as a powerful endogenous angiogenesis inhibitor, but its role in macrophage recruitment and polarization is largely unknown. Gao et al. [40] found that PEDF dampened neovascularization by mediating macrophage recruitment and polarization in the mouse model of oxygen-induced retinopathy (OIR). The macrophage polarization was mediated by regulating the phosphorylation of MAPKs, including p38 MAPK, Jun N-terminal kinase (JNK), extracellular regulated protein kinases (ERK), and Notch1 accumulation through its receptor adipose triglyceride lipase (ATGL).

### 3.3. Inhibition of Neovascular Permeability

Vascular permeability plays a key role in the sight-threatening diseases. VEGF promoted angiogenesis and vascular permeability. PEDF is not only an antiangiogenic agent; it also inhibits pathologically increased vascular permeability. Liu et al. [41] found that PEDF effectively abated VEGF-induced vascular permeability by using a model system for nonproliferative diabetic retinopathy (NPDR). They suggested that PEDF might be an important therapeutic adjunct in the treatment of vascular permeability-induced sight-threatening diseases.

Studies have shown that p38 MAP kinase and ERK play a role in VEGF-induced vascular hyperpermeability [42, 43]. Yang et al. studied the mechanism by which PEDF blocks VEGF-induced increases in vascular permeability. They found that PEDF prevented VEGF-induced decreases in endothelial cell transendothelial electrical resistance (TER). They first showed that PEDF blocked phosphorylation/activation of p38 MAP kinase and ERK in a way that was comparable with the effects of specific inhibitors of p38 mitogen-activated protein (MAP) kinase and extracellular regulated protein kinases (ERK) [44]. PEDF is naturally present in the eye in significant quantities, and thus, these activities may help maintain the normal tight blood-retinal barrier of the eye. Another study found that PEDF blocks VEGF-induced vascular permeability by preventing dissociation of endothelial adherens junctions (AJs) and tight junctions (TJs) via a *γ*-secretase-dependent mechanism. They suggested that targeting downstream effectors of PEDF, such as VEGFR translocation and/or *γ*-secretase activity, may represent a promising therapeutic strategy for the treatment of diabetic vascular complications in the eye [45].

Yamagishi et al. investigated how PEDF could inhibit the advanced glycation end product (AGE) signaling to vascular hyperpermeability. They demonstrated that the central mechanism for PEDF inhibition of the AGE signaling to vascular permeability is by suppression of nicotinamide adenine dinucleotide phosphate (NADPH) oxidase-mediated reactive oxygen species (ROS) generation and subsequent VEGF expression [46]. Also, PEDF inhibited the AGE-induced upregulation of VEGF mRNA levels in cultured endothelial cells.

## 4. PEDF in Choroidal Neovascularization (CNV)

PEDF has antiangiogenic and neuroprotective functions, and many studies have tried to use PEDF to treat CNV animal models. Mori et al. found that intravitreous injection of an adenoviral vector encoding PEDF (AdPEDF) could promote the expression of PEDF mRNA in the eye, which could be measured by Reverse Transcription-Polymerase Chain Reaction (RT-PCR) and immunohistochemical staining for PEDF protein throughout the retina. Then, they tried the intravitreous or subretinal injection of AdPEDF.10 in mice with laser-induced CNV, mice with VEGF-induced neovascularization, and mice with ischemia-induced retinal neovascularization. They suggested that PEDF gene transfer by intraocular injection of PEDF-containing vectors might provide a promising approach for treatment of ocular neovascularization [47]. Another experiment tested the effect of periocular injection of an expression cassette for PEDF packaged in adenoviral vector (AdPEDF.11) in a CNV model in pigs. They found that a sufficient increase in PEDF in the choroid could be tested after periocular injection of AdPEDF.11, and the periocular injection of AdPEDF.11 could suppress CNV [48].

An adeno-associated viral (AAV) vector containing expression construct coding for PEDF could inhibit CNV [49]. Intraocular expression of PEDF or other antiangiogenic proteins with AAV vectors may provide a new treatment approach for ocular neovascularization. Hamilton et al. used Ad35-based vectors, which could express PEDF in the mouse model of laser-induced CNV by intravitreal (IVT) administration. They found that compared to an Ad5-based vector, the gene expression was prolonged through administration of Ad35. Also, PEDF expression from an Ad35.PEDF (HI-RGD) vector was able to inhibit CNV lesion growth by more than 80% at day 42 as compared to the no-injection control [50]. Askou et al. investigated whether dual-acting therapy based on the simultaneous expression of anti-VEGF and PEDF delivered by AAV vectors provides improved protection against laser-induced CNV in a mouse model. CNV reduction was most prominent in a CNV mouse model receiving dual-acting therapy [51]. They suggested that dual-acting therapy by gene therapy might be an important tool in the treatment of neovascular ocular diseases, including AMD. Park et al. generated PEDF transgenic (PEDF-Tg) mice that ubiquitously express human PEDF driven by the *β*-actin promoter. They found that overexpression of PEDF inhibits laser-induced CNV in mouse models [52].

The sclera and choroid-RPE are permeable to PEDF and ovalbumin proteins, and these proteins can traverse the subconjunctiva to reach the retina [53]. Subconjunctival protein delivery might represent a feasible and minimally invasive route for PEDF administration. Amaral and Becerra examined the effects of recombinant human PEDF (rhuPEDF) and PEDF-derived peptide 34-mer on vessel sprouting and on CNV after subconjunctival administration in laser-induced rats. The rhuPEDF and PEDF-derived peptide 34-mer inhibited vessel sprouting in a dose-response fashion. Also, a functional region for the inhibition of vessel sprouting and CNV resided within the 34-mer region of PEDF. This meant that the agents such as rhuPEDF and 34-mer could reach the choroid-RPE complex as functional active molecules, and subconjunctival administration of optimal range dosages of rhuPEDF or 34-mer could suppress rat CNV lesions [54].

Lentivirus-mediated transfer of PEDF was shown to be effective for treating laser-induced CNV. Yu et al. evaluated the effects of lentivirus-mediated PEDF gene transfer in the treatment of laser-induced rat CNV. They found that lentiviral vectors appeared to be safe and effective for gene expression, with little immune response by the recipient [55]. A lentiviral vector (LV) delivered the expression of therapeutic anti-VEGF-miRNAs and PEDF from a single cassette and efficient gene transfer to mouse retinal cells, demonstrating the potential therapy for exudative AMD [56].

Semkova et al. generated a high-capacity adenovirus (HC-Ad) vector expressing human PEDF, transduced cultured iris pigment epithelial (IPE) cells. They transplanted these modified cells to the subretinal space of laser-induced CNV rats and found that they inhibited neovascularization and improved the survival of photoreceptors [57].

## 5. Protective Effect of PEDF

### 5.1. Protective Effect of PEDF on RPE

The functional response of RPE cells to PEDF is polarized to the apical side. The PEDF secretion pattern from the RPE cells is predominantly apical [58]. VEGF release is higher basally than apically, and PEDF release is higher apically than basally [59].

The protective effect of PEDF on the barrier function of the RPE and the H_2_O_2_-induced RPE monolayer permeability was examined. The results showed that PEDF could prevent H_2_O_2_-induced RPE permeability changes and preserve the barrier function of RPE cells against oxidative stress [60]. H_2_O_2_-induced redistribution of the junctional proteins and actin reorganization were decreased by using PEDF. He et al. [61] investigated the role of PEDF in limiting oxidative stress-induced damage to RPE cells through mitochondrial pathways. They found that PEDF could reverse H_2_O_2_-induced RPE cell death and oxidative stress disruption of mitochondrial reticular networks. Also, they found that an increasing PEDF dose had an overall slightly better effect on mitochondrial function. So, a possible mechanism for PEDF's neuroprotective actions might be through restoring mitochondrial dynamics perturbed by both aging and oxidative stress conditions. Subramanian et al. evidenced that PEDF-R protects RPE cells from oxidative stress-induced cell death via inhibiting 5-lipoxygenase (5-LOX) [62]. PEDF-R peptides E5b and P1 are inhibitors of LOX. This suggested a possible therapeutic approach for RPE under oxidative stress conditions.

It was reported that PEDF protects RPE cells from toxic damage. Nadal-Nicolas and Becerra induced cytotoxicity injury to ARPE-19 cells with NaIO_3_ and established an *in vitro* model system to study RPE degeneration and protection. They found that the cytotoxicity could be decreased by using PEDF. PEDF decreased ARPE-19 cytotoxicity with 6 mM and 7 mM NaIO_3_ but did not increase the cell viability in response to 6-8 mM NaIO_3_ [63].

### 5.2. Protective Effect of PEDF on Photoreceptor Cells

The mechanism by which PEDF protects photoreceptor cells is not clear. Wang et al. evaluated the neuroprotective and anti-inflammatory effects of PEDF on the retinal lesions in a DKO rd8 mouse model of progressive, focal retinal degeneration, mimicking certain features of human atrophic AMD. They found that PEDF potently stabilizes photoreceptor degeneration via suppression of both the apoptotic and inflammatory pathways [64]. One study found that increased aging and stress reduce activation of the PI3K/Akt and MAPK pathways in RPE cells and the actions of PEDF on mitochondrial dynamics are mediated, in part, through the PI3K/Akt pathway [61]. This means that a possible mechanism for PEDF's neuroprotective actions is through restoring mitochondrial dynamics perturbed by both aging and oxidative stress conditions. Comitato et al. explored *in vitro* and *in vivo* the role of PEDF on the extrusion of calcium using specific Ca^2+^ pump inhibitors in models of rd1 mice. They found that PEDF acted on PMCA to reduce intracellular calcium at early stages of the rod photoreceptor cell death cascade. A decrease in the number of photoreceptors with high intracellular Ca^2+^ was achieved after treatment with PEDF by restraining calpain activation. PEDF protects the degenerating retina by decreasing intracellular calcium [65]. The results defined that PEDF neuroprotection acted on the cell death pathway activated by calcium influx and mediated by apoptosis-inducing factor (AIF) activation in photoreceptor cells.

Cao et al. demonstrated that the intravitreal injection of PEDF at one day or two days before exposure to constant light could protect photoreceptor cells from damage. When PEDF was used in combination with the basic fibroblast growth factor (bFGF), there was an enhancement of the functional status of the photoreceptor cells beyond the protection provided by the factor alone whereas PEDF given at, or after, the onset of constant light provided little or no protection [66]. They suggested that these two factors together could be useful for the development of strategies for treatment and prevention of blindness due to a variety of causes. Cayouette et al. investigated whether PEDF has survival-promoting activity on degenerating photoreceptors *in vivo* by using the retinal degeneration (rd) and retinal degeneration slow (rds) mutant mouse models. They found that a significant decrease in the death of photoreceptors was caused by injecting the purified protein into the vitreous body of the eye in the two mutants [67]. Their results suggested that PEDF could be required for the normal development and survival of photoreceptors.

PEDF may offer new therapeutic strategies in the treatment of retinal detachment and other degenerations that are induced by dystrophies of the RPE [68]. The expression of PEDF was significantly decreased in Mitf-deficient RPE. Chen et al. recombined the mutant-derived postnatal retina with postnatal wild-type RPE in tissue explant cultures. They found that when the Mitf^−/−^ retina was reconstituted with WT RPE, rhodopsin expression and outer nuclear layer (ONL) thickness were partially restored [69]. Also, they tested whether PEDF might have a similar rescue effect *in vivo* by using eye drops containing a small peptide fragment, PEDF 17-mer. The results showed that application of the PEDF 17-mer peptide enhanced photoreceptor survival and partially prevented retinal degeneration in Mitf mutant mice.

### 5.3. Protective Effect of PEDF on Ganglion Cells

PEDF protects nerves by different mechanisms. One study reported that the neuroprotective effect of PEDF is associated with both antiapoptotic and anti-inflammatory activities on retinal neuronal cells [64]. The preoptimised doses of PEDF and PEDF-34 *in vitro* and *in vivo* caused significant and specific reduction in caspase 2 (CASP2) mRNA and protein in retinal ganglion cells (RGC), and RGC neuroprotection is proportional to suppressed CASP2 levels [70].

Müller cell-derived PEDF could protect RGC from hypoxia and growth factor withdrawal-induced cell death. The number of viable retinal ganglion cells (RGC) increases when cocultured with Müller glial cells, which could release PEDF [71]. Vigneswara et al. investigated neuroprotective and axogenic properties of PEDF in retinal ganglion cells (RGC) *in vitro* and *in vivo*. They confirmed previous reports that PEDF promoted RGC survival *in vitro* and *in vivo*. Also, they demonstrated for the first time that PEDF promoted RGC neurite outgrowth/axon regeneration. Combining PEDF with cAMP did not enhance PEDF-induced RGC neuroprotection but did enhance RGC axon regeneration [72].

### 5.4. Protective Effect of PEDF on Pericytes

Microvessels are composed of two types of cells, endothelial cells (ECs) and pericytes. Pericytes control the overgrowth of neighboring ECs under physiological conditions and play an important role in the maintenance of microvascular homeostasis. Pericytes tighten the tight junction of ECs to reduce endothelial permeability. They play an important role in maintaining the integrity of the inner blood-retinal barrier (BRB) by surrounding ECs [73]. One study found that the pericyte survival increases and the vascular permeability observed in the diabetic retinas of mice decreases [74]. Pericytes may strengthen the tight junction of the ECs.

Oxidative stress-induced apoptosis and increased ratio of angiopoietin-2/angiopoietin-1 in pericytes could disrupt the pericyte-endothelial cell interactions, thus promoting angiogenesis by inducing VEGF mRNA levels in retinal pericytes. Amano et al. found that PEDF could protect retinal pericytes against high-glucose or H_2_O_2_-induced apoptosis and dysfunction through its antioxidative properties via glutathione peroxidase (GPx) [75]. Oxidative stress has been implicated in triggering apoptosis. Antiapoptotic effects of PEDF observed in various cell types could be ascribed to its antioxidative activities.

Yamagishi et al. investigated whether PEDF proteins could protect against advanced glycation end product- (AGE-) induced injury in retinal pericytes. They found that a specific PEDF binding protein existed in the plasma membrane of cultured pericytes, suggesting a possible cell surface receptor for PEDF in its inhibition of AGE-induced reactive oxygen species (ROS) generation and the subsequent decrease in DNA synthesis and apoptotic cell death in pericytes [76].

## 6. Antifibrotic Effect of PEDF

### 6.1. PEDF and Extracellular Matrix

PEDF could bind to extracellular matrix (ECM) components such as collagens and glycosaminoglycans (GAGs). The different biological activities of PEDF could be based on the interaction with collagens and GAGs.

Collagen is the predominant extracellular matrix protein which plays an important role in cell adhesion and migration. The PEDF recognizes collagen in a sequence- and conformation-specific manner [77]. Yasui et al. found that PEDF possessed dual binding sites for different ECM components. The acidic amino acid residues on the PEDF are necessary for collagen binding, and three clustered basic amino acid residues contribute to heparin binding [78].

At the molecular level, PEDF has binding sites for collagens and glycosaminoglycans. The collagen-binding property of PEDF may play a role in modifying its antiangiogenic effects in the eye [79]. The association of PEDF with the extracellular matrix such as glycosaminoglycan is mediated by ionic interactions. The glycosaminoglycan-binding site can be identified on PEDF. This binding represents the likely molecular basis for its association with the extracellular matrix [80].

### 6.2. The Mechanism of Antifibrotic Effect of PEDF

Vascular changes often accompany the development of fibrosis. Transforming growth factor-*β* (TGF-*β*) has been linked to the development of fibrosis in a number of diseases, and tissue fibrosis is primarily attributable to the TGF-*β*1 isoform [12]. Circulating monocytes and macrophages are the predominant cellular source of TGF-*β*1. Macrophage-derived TGF-*β*1 is thought to promote fibrosis by directly activating resident mesenchymal cells including epithelial cells.

Wang et al. injected streptozotocin- (STZ-) induced diabetic rats with an adenovirus expressing PEDF (Ad-PEDF) to evaluate its effects in diabetes. Administration of Ad-PEDF could prevent the overexpression of fibrogenic factors, TGF-*β*1 and connective tissue growth factor (CTGF). Also, it reduced the production of extracellular matrix (ECM) protein production in a diabetic kidney [81]. This means that PEDF could suppress mesangial matrix accumulation through the blockade of hyperglycemia-induced TGF-*β*1 and CTGF expression. They suggested that the reduction of ECM accumulation induced by PEDF might be mediated in part by the downregulation of TGF-*β*1.

TGF-*β* has been recognized as a modulator of ECM formation, such as fibronectin and collagen. TGF-*β* is a major pathogenic factor in diabetic nephropathy, and it is increased under high-glucose conditions. One study found that PEDF blocked high glucose-induced TGF-*β* overexpression in primary human glomerular mesangial cells (HMCs) [82].

Endothelial mesenchymal transition (EndMT) plays a critical role in the pathogenesis and progression of interstitial and perivascular fibrosis after acute myocardial infarction (AMI). Zhang et al. reported that endogenous PEDF expression could inhibit cardiac fibrosis especially perivascular cardiac fibrosis by regulating EndMT via downregulating the transcriptional activation of *β*-catenin in an ischemic heart [83].

Wnt signaling promotes inflammation and oxidative stress. He et al. found that PEDF knockout enhanced unilateral ureteral obstruction- (UUO-) induced Wnt signaling activation, and PEDF inhibited the Wnt pathway-mediated fibrosis in renal proximal tubule epithelial cells [84]. They suggested that the antifibrotic and antioxidative effects of PEDF were mediated in part by inhibiting Wnt signaling.

### 6.3. Application of PEDF against Fibrosis

Many experiments have tried to examine the antifibrotic effect of PEDF. However, no application of PEDF antifibrosis in ophthalmology has been found.

Tsai et al. found that the amount of PEDF was reduced dramatically in the fibrotic liver. The antifibrotic effect of PEDF is preserved in its 34-mer motif, and its antifibrotic activity was confirmed in an *in vivo* mouse model of carbon tetrachloride- (CCl_4_-) induced hepatic fibrosis [85]. So, the PEDF-derived short peptide might be used as an antifibrotic agent for treating liver fibrosis.

Inflammatory response can lead to increased collagen deposition and fibrosis [12]. Principe et al. demonstrated that PEDF limited pancreatic cancer progression by attenuating the fibroinflammatory reaction [9]. Also, they found that increased fibrosis seen in the absence of PEDF in mice or humans was associated with enhanced transforming growth factor-*β*1 (TGF-*β*1) expression. PEDF treatment could significantly attenuate VEGF expression and might lead to the inhibition of airway inflammation and airway remodeling [10]. Ho et al. demonstrated that PEDF was an intrinsic antifibrotic factor that prevented hepatic stellate cell (HSC) activation via induction of peroxisome proliferator-activated receptor gamma (PPAR*γ*) [11]. They observed that exogenous PEDF can prevent chemically induced liver fibrosis in mice.

## 7. The Possible Role of Polymorphisms in the PEDF Gene in Wet AMD Pathogenesis

Family history has a significant association with the incidence of AMD [86], which suggests that genetic factors may be related to the occurrence of AMD. A significant association between two major risk alleles, complement factor H (CFH) and age-related maculopathy susceptibility 2 (ARMS2) rs10490924, and the incidence of AMD in Ireland have been reported [87]. To date, the role of polymorphisms in the PEDF gene in wet AMD pathogenesis has been investigated. However, there has been no definitive conclusion.

The relationship between PEDF polymorphisms and the risk of AMD in northern Chinese populations has been studied. The genotype and allele distribution of rs1136287 polymorphism were different between AMD patients and healthy people [88]. The rs1136287 polymorphism in PEDF may be related to the occurrence risk of AMD. Another study came to the same conclusion. PEDF-rs1136287 variants were associated with visual outcomes in response to intravitreal bevacizumab treatment for AMD. Genetic polymorphisms might be utilized as genetic biomarkers to predict the outcome after treatment in AMD [89]. Lin et al. investigated the Met72Thr (T/C) polymorphism (rs1136287) of the PEDF gene and found that the homozygous T genotype was more prevalent in wet AMD than in controls. They suggested that the PEDF methionine-to-threonine polymorphism (Met72Thr) T allele may be a risk factor for wet AMD in the Taiwan Chinese population [90]. A novel hypothesis is that the Met72Thr polymorphism (T/C polymorphism) of the PEDF gene may be associated with the susceptibility to AMD and may be a genetic marker for AMD [91].

Mattes et al. investigated a hypothesized association between these PEDF polymorphisms and the presence of exudative AMD in a white European population. They found that none of the investigated PEDF polymorphisms (rs12150053 and rs12948386) were likely a major risk factor for exudative AMD. Two polymorphisms did not confer a significantly increased risk for exudative AMD [92]. TaqMan technology was used to genotype the Met72Thr variant (rs1136287). However, there was a lack of association between the PEDF Met72Thr variant and either neovascular AMD or PCV in a Japanese population [93]. They concluded that the Met72Thr variant does not play a significant role in the risk of developing neovascular AMD or PCV. Also, detection of two common nucleotide polymorphisms (SNPs), Met72Thr (rs1136287) and -5736T>C (rs12150053), in the PEDF gene was not found to be significantly associated with exudative AMD in the Chinese cohort [94]. A meta-analysis of four PEDF polymorphisms, rs1136287, rs12150053, rs12948385, and rs9913583, was not significantly associated with AMD [95].

The etiology of AMD is multifactorial, with genetic risk factors contributing to the disease development and progression. Further studies on the role of PEDF gene variations in the pathogenesis of wet AMD are required.

## 8. PEDF Levels in Patients with CNV Secondary to Pathological Myopia

Myopia, especially high myopia, can lead to permanent loss of vision. CNV incidence increases in patients with high myopia. PEDF levels in the aqueous in high myopia patients have been investigated in many studies. But different studies come to different conclusions. Costagliola et al. investigated the VEGF and PEDF levels in patients with CNV secondary to pathological myopia (mCNV). Comparing with control samples, significantly lower levels of VEGF and PEDF in the aqueous humor were found in patients with mCNV. After intravitreal ranibizumab injection (IVR), the aqueous VEGF level was significantly reduced, but the PEDF level was significantly increased. An increase in PEDF levels may counteract the neovascularization process in mCNV [96]. However, another study reported that aqueous humor levels of VEGF were lower in the high myopia group than in the control group. But PEDF levels tended to be higher in the high myopia group compared to the control group, especially in high myopia with posterior staphyloma [97]. These results suggested that degeneration of RPE cells in high myopia might influence the secretion of VEGF, and high myopia with posterior staphyloma might disrupt the balance of VEGF and PEDF. One study found that aqueous levels of PEDF were significantly higher in high myopia with CNV patients than non-CNV patients, and there was a possible correlation between aqueous PEDF and macular choroidal thickness in the non-CNV myopia patients [98]. This means that in vivo macular choroidal thickness may be indicative of aqueous PEDF concentration in high myopia with no CNV.

The PEDF levels in Tenon's capsule of myopes were investigated. The level of “soluble” 45 kDa PEDF was reduced by twofold in Tenon's capsules of high myopia patients while the content of the “insoluble” 50 kDa form of PEDF was increased by fourfold [99]. This result indicated that abnormal metabolism rather than the decrease in PEDF synthesis might be correlated with myopia development. The genetic background of the occurrence of CNV secondary to high myopia was explored [100]. There might be a possible association between the PEDF SNP rs12603825 and the occurrence of myopic CNV. But the result lacked significance after multiple comparisons.

## 9. Conclusions and Future Directions

PEDF is naturally present in the eye, and it not only functions as an antiangiogenic and neurotrophic/neuroprotective agent but also inhibits pathologically increased vascular permeability. The role of PEDF in antiangiogenesis and protection of pericytes can be applied to the treatment of diabetic retinopathy and CNV in the future. Also, the neurotrophic/neuroprotective effect of PEDF is more advantageous than single anti-VEGF drugs. PEDF inhibits the VEGF-induced barrier breakdown of RPE cell monolayers through the activation of *γ*-secretase. So, a better understanding of the action of PEDF may provide a more effective therapeutic approach to the treatment of VEGF-induced pathogenesis in the eye. Furthermore, combined anti-VEGF and PEDF may play a synergistic role in the treatment of CNV. However, the potential toxicities or side effects caused by PEDF monotherapy or combination therapy with anti-VEGF need further investigation. The antioxidative effect of PEDF is promising as a way to treat retinal degenerative diseases. PEDF has an anti-inflammatory action, and whether it can suppress inflammation occurring in the retina and reduce macrophage infiltration and fibrosis needs to be investigated in the future. The role of PEDF in the eye is not clear, and further research is needed.
